# Food Safety and Natural Toxins

**DOI:** 10.3390/toxins12040236

**Published:** 2020-04-08

**Authors:** Mary T. Fletcher, Gabriele Netzel

**Affiliations:** Queensland Alliance for Agriculture and Food Innovation (QAAFI), The University of Queensland, Health and Food Sciences Precinct, Coopers Plains, QLD 4108, Australia; g.netzel@uq.edu.au

Natural toxins are poisonous secondary metabolites produced by living organisms, which are typically not harmful to the organisms themselves but can impact on human or animal health when consumed [1,2]. Common sources of such toxins include poisonous plants, fungi, algae, bacteria and marine biotoxins, and the diversity of these biological systems presents challenges to analytical chemists and wide-ranging food safety implications. The propensity for such toxins to be present in both animal feed and human food has led to the introduction of regulations for a small number of the most potent natural toxins, particularly mycotoxins [3]. Implementation of these regulations necessitates the establishment of high-throughput analytical chemistry methods [4], such as LC-MS and GC-MS, with increasingly lower limits of detection, and the production of isotopically labelled analogues as internal standards to increase the reliability of analysis in complex matrices. Thus, this Special Issue focused on the analysis of natural toxins, their incidence from source organisms to food and feed commodities, and implications for food safety.

The impacts of natural toxins present in pasture plants are not limited to grazing livestock but can also be carried through the food chain, with some toxins, such as indospicine, being demonstrated to accumulate in the tissues of grazing animals and cause secondary poisoning in animals consuming meat from these livestock [5,6]. Interestingly, camel feeding trials have demonstrated that indospicine is still detectable by LC-MS/MS analysis 100 days after cessation of feeding *Indigofera* plants, whereas ruminal metabolites of this toxin have a considerably faster clearance rate [5]. The toxicity of indospicine for humans is uncertain but simple cooking of indospicine-contaminated meat does not degrade this natural toxin, and in vitro studies under human gastrointestinal conditions further demonstrated its stability and release into liquid digesta indicating ready bio-accessibility for absorption in the small intestine [6]. 

Mycotoxins are natural toxins produced by fungi in a range of food and feed commodities, with potent carcinogenic aflatoxins (particularly aflatoxin B_1_ or AFB1) being considered of significant risk to human health. Once ingested by animals, AFB1 can be carried into milk as the toxic metabolite aflatoxin M_1_, with the presence of AFM1 in milk being of particular concern as young children represent a particularly susceptible age group [7]. AFM1 analysis studies have assessed the risk of AFM1 in both liquid and powdered milk marketed in Pakistan, with processed milk shown to have lower AFM1 levels than raw milk [7]. Mycotoxin risk is not only limited to the consumption of cereal grains, but can also occur in pollen as used in food supplements [8], and malting and brewing by-products as used in animal feed [9], and reviews of these products further highlight the potential mycotoxin risks in these novel food and feed products.

Natural toxins produced by bacteria are also of concern. Shiga toxin-producing *Escherichia coli* is a frequent cause of food poisoning with enormous human health and economic impacts, and rapid and accurate identification is imperative for protection of human health. Detection of the toxin by a newly developed amplified luminescent proximity homogenous assay-linked immunosorbent assay (AlphaLISA) allows for an automated and much more rapid assay of the toxin with increased sensitivity and dynamic range compared to the industry-standard ELISA test [10].

Another class of natural toxins are ciguatoxins (CTXs), marine biotoxins produced by dinoflagellates of the genus *Gambierdiscus*, with the foodborne disease ciguatera caused by the consumption of contaminated seafood. Analytical studies have examined both CTXs’ tissue distribution in giant clams (*Tridacna maxima*) exposed to toxic cells of *Gambierdiscus polynesiensis*, and also detoxification rates after the transfer of clams into clean water [11]. Viscera, flesh, and mantle contained 65%, 25%, and 10% of the toxin burden respectively, with all tissues reaching levels above safety limits, and most concerningly, no toxin elimination was seen within a six day detoxification period.

This Special Issue then contains original contributions that advance our knowledge of the food safety implications of natural toxins. The breadth of the manuscripts demonstrated the diverse sources of natural toxins that can affect food safety, from plant toxins and their accumulation in the meat of grazing livestock, mycotoxins and their presence in grains, brewing by-products, milk and pollen supplements, ciguatoxins in seafood, and Shiga toxins associated with *Escherichia coli* contamination of foods.
